# Electrochemical sensor based on α-Fe_2_O_3_/rGO core-enhanced carbon interfaces for ultra-sensitive metronidazole detection

**DOI:** 10.1038/s41598-025-19377-w

**Published:** 2025-10-09

**Authors:** Abdulrahman Saad Alqahtani, Hashim Elshafie, Azath Mubarakali, Asma AlJarullah, P. Parthasarathy, M. Venkatesh, S. Adarsh Rag

**Affiliations:** 1 College of Computing and Information Technology, University of Bisha, Bisha, 67714 Saudi Arabia; 2 Department of Computer Engineering, College of Computer Science, King Khalid University, Abha, 61421 Saudi Arabia; 3 Department of Informatics and Computer Systems, College of Computer Science, King Khalid University, Abha, 61421 Saudi Arabia; 4 Department of ECE, CMR Institute of Technology, Bengaluru, 560037 Karnataka India; 5 Department of Data Science and Computer Applications, Manipal Institute of Technology, Manipal Academy of Higher Education, Manipal, 576104 Karnataka India

**Keywords:** Magneto-electrochemical biosensor, α-Fe_2_O_3_/Reduced graphene oxide (rGO), Metronidazole detection, Electrocatalytic activity, Pharmaceutical and clinical diagnostics, Biological techniques, Nanoscience and technology

## Abstract

In this work, we describe the creation of a new magneto-electrochemical biosensor that detects metronidazole (MTZ), an antibiotic that is frequently used to treat anaerobic bacterial and protozoal infections, with extreme sensitivity. The sensor platform is engineered by integrating α-Fe_2_O_3_ magnetic core nanoparticles with reduced graphene oxide (rGO) to fabricate a core-enhanced carbon electrode (α-Fe₂O₃/rGO@CE). The synergistic combination of α-Fe_2_O_3_ and rGO significantly enhances the electrocatalytic activity, electron transfer rate, and surface area of the sensing interface. Using X-ray diffraction (XRD), electrochemical impedance spectroscopy (EIS), and scanning electron microscopy (SEM), structural and morphological characterizations were carried out to verify the uniform distribution of spherical α-Fe_2_O_3_ nanoparticles (~ 25 nm) anchored on rGO nanosheets. Electrochemical performance was systematically investigated through cyclic voltammetry (CV) and Differential Pulse voltammetry (DPV). When compared to the unmodified Counter Electrode (CE) (-0.65 V against Ag/AgCl), the suggested biosensor showed a notable change in the metronidazole reduction peak to a higher positive potential (-0.4 V vs. Ag/AgCl), suggesting superior catalytic efficiency. With a remarkable limit of identification (LOD) of 2.80 × 10^−6^ M and a limit of quantization (LOQ) of 8.0 × 10^−6^ M, a broad linear detection range of 8.0 × 10^−6^ to 1.0 × 10^−5^ M was attained. The sensor was effectively used for the quantitative measurement of metronidazole in medication and in human urine samples (collected from Mangalore Medical Centre with informed consent obtained from the respective patients, ensuring ethical compliance for clinical analysis) due to its exceptional sensitivity, stability, and reproducibility. This study demonstrates how α-FeO₃/rGO hybrid nanomaterials can be used to create effective magneto-electrochemical biosensors for use in clinical and pharmaceutical diagnostic settings.

## Introduction

**Fig. 1:**
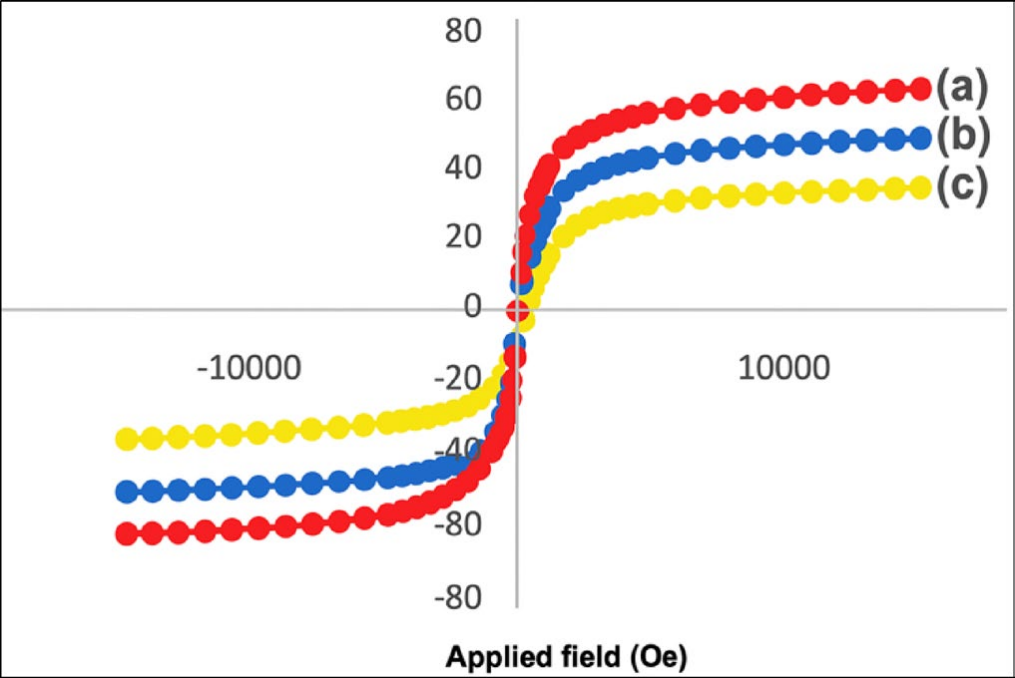
Vibrating sample magnetometer (VSM) curves of (**a**) α-Fe_2_O_3_/rGO, (**b**) Fe_2_O_3_, (**c**) rGO.

Metronidazole (MTZ), chemically known as 2-methyl-5-nitroimidazole-1-ethanol, is a widely prescribed antibiotic for the treatment of anaerobic bacterial and protozoal infections, such as amoebiasis, giardiasis, trichomoniasis, and bacterial vaginosis. Apart from its widespread application in human health, MTZ is also used as a growth-promoting ingredient in the aqua-culture sector and in veterinary medicine to prevent and treat infections. Despite its therapeutic benefits, excessive exposure to metronidazole can lead to adverse effects, including neurotoxicity, characterized by convulsions, ataxia, and potential genotoxicity^1^. Studies have demonstrated MTZ’s ability to penetrate bacterial cell membranes, disrupt DNA synthesis, and inhibit the replication of various microorganisms, raising concerns over its genotoxic and mutagenic potential in both in vivo and in vitro models. Given the potential risks associated with metronidazole overuse and residue accumulation in biological and environmental systems, the development of a reliable, sensitive, and selective detection method is critically important. Accurate quantification of MTZ is essential not only for pharmaceutical quality control but also for monitoring its presence in environmental matrices such as tap water and biological fluids, including urine. Metronidazole has been determined using a number of traditional analytical methods, such as amperometric colorimetry, nuclear magnetic resonance spectroscopy, shortwave near-infrared spectroscopy, spectrophotometry, high-performance liquid chromatography, and HPLC-mass spectrometry. While these methods offer high precision and specificity, they often suffer from limitations such as the requirement for expensive instrumentation, complex sample preparation procedures, and the extensive use of high-purity organic solvents. These constraints render them less accessible, time-consuming, and cost-prohibitive for routine and on-site analysis^2,3^. Electrochemical sensing platforms offer a cost-effective, sensitive, and rapid approach for detecting pharmaceutical compounds like metronidazole (MTZ). Incorporating nanomaterials such as magnetic metal oxides and carbon-based nanostructures enhances their electron transfer efficiency and active surface area, improving analytical performance. Modified electrodes, including zinc oxide carbon paste and ferrite nanoparticle-based sensors, have demonstrated excellent sensitivity for detecting MTZ, which possesses a reducible nitro group. Techniques like square wave voltammetry (SWV) and differential pulse voltammetry (DPV) further enhance detection sensitivity and selectivity. Among nanomaterials, iron oxide-based magnetic nanoparticles stand out for their conductivity, stability, and abundance of active sites in pharmaceutical sensing. Although bulk α-Fe₂O₃ (hematite) is typically weakly antiferromagnetic at room temperature, the α-Fe₂O₃ nanoparticles (~ 25 nm) used in this work exhibit size-induced surface spin canting, leading to weak ferromagnetism or superparamagnetic behavior. This was confirmed by vibrating sample magnetometry (VSM) as our preliminary study, where the α-Fe₂O₃/rGO hybrid displayed a saturation magnetization (Ms) of approximately 3.4 emu/g with negligible coercivity (Fig. 1). While magnetic properties were not directly exploited during electrochemical measurements, they reflect the nanostructured morphology and surface-active characteristics of the material, which synergistically enhance electron transfer and electrocatalytic efficiency.

In this study, we report the fabrication of a magneto-electrochemical biosensor based on α-Fe₂O₃/reduced graphene oxide (rGO) core-enhanced carbon interfaces (α-Fe₂O₃/rGO@CE) for ultra-sensitive detection of metronidazole. The synergistic integration of spherical α-Fe₂O₃ nanoparticles (~ 25 nm) with rGO nanosheets significantly improves the electrode’s surface area, electron transfer kinetics, and electrocatalytic activity. The α-Fe₂O₃/rGO hybrid nanomaterial not only provides excellent stability and durability but also enhances the sensor’s sensitivity toward MTZ detection in complex media. SEM (scanning electron microscopy) and X-ray diffraction (XRD) were used to confirm the microscopic and morphological characteristics of the α-Fe₂O₃/rGO@CE sensor. The results showed that the α-Fe₂O₃ nanoparticles were uniformly distributed over the rGO sheets. In comparison to the unaltered carbon electrode, electrochemical evaluation using cyclic voltammetry (CV) and DPV showed a notable reduction in charge transfer barrier and an improved current responsiveness. Additionally, the biosensor addressed the urgent need for a straightforward, quick, and accurate analytical tool by demonstrating exceptional sensitivity, rigidity, and accuracy in the quantitative detection of MTZ in drugs and human urine samples^4–6^.

## Materials and methods

Every chemical and reagent utilized in this investigation was analytical grade and didn’t require any additional purification. Double-distilled water was used in the preparation of all stock and solutions of electrolytes. Carbon graphite (CG) and the active medicinal element metronidazole (MTZ) were acquired from KSC Chemicals in Chennai. Additional chemicals were purchased from Sigma-Aldrich, including potassium chloride (KCl), sulfuric acid (H₂SO₄), ferric chloride (FeCl₃), and iron (III) nitrate nonahydrate (Fe (NO₃) ₃·9 H₂O). KSC Chemicals supplied the potassium hexacyanoferrate (III) (K₃Fe (CN)₆) and potassium hexacyanoferrate (IV) (K₄Fe (CN)₆) that were utilized as redox probes. By combining 0.15 M K₂HPO₄ and 0.15 M KH₂PO₄ solutions, phosphate buffer solutions (PBS) with different pH values were created. Concentrated H₂SO₄ or NaOH were used as needed to modify the pH. Strict quality control procedures were used for solution preparation and pH changes^7,8^.

### Synthesis of α-Fe_2_O_3_/rGO nanocomposite

**Fig. 2:**
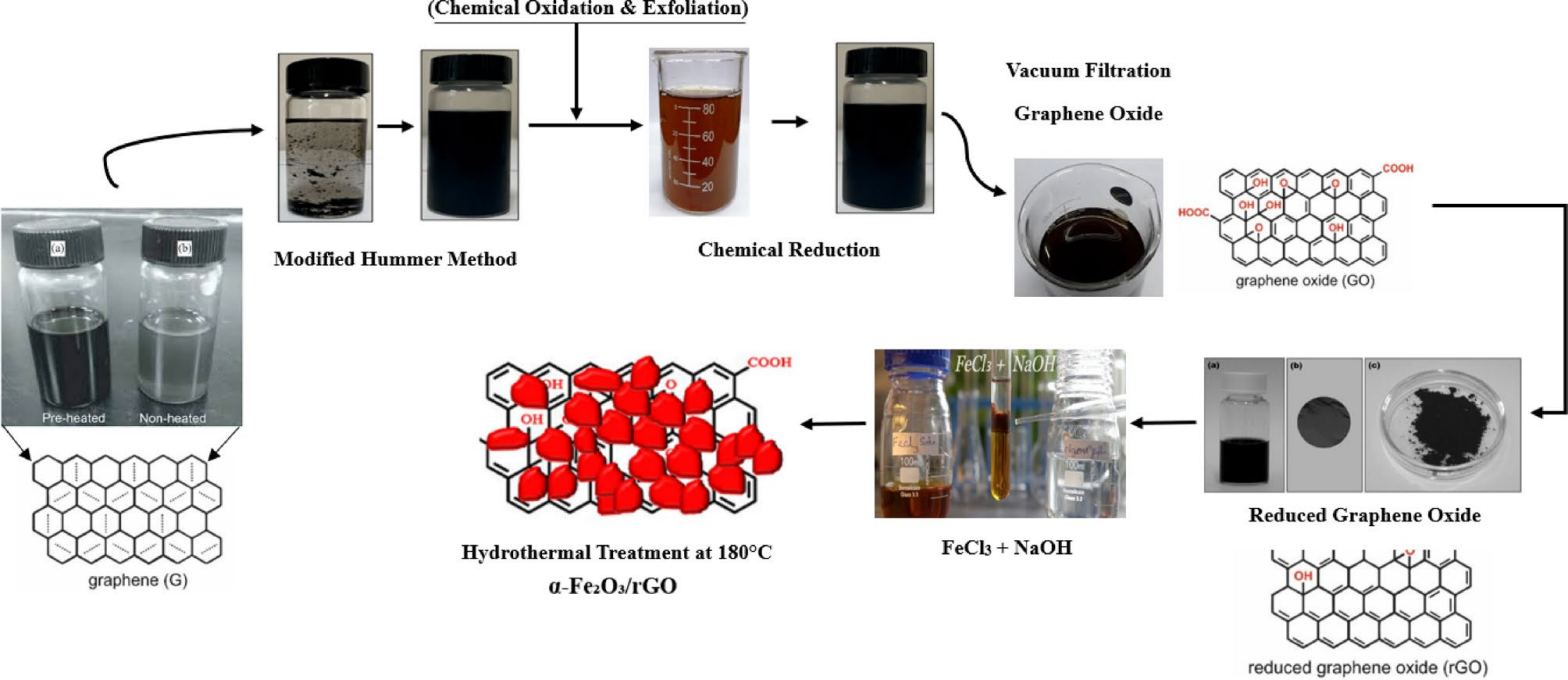
Synthesis of α-Fe_2_O_3_/rGO nanocomposite.

The overall Synthesis of α-Fe₂O₃/rGO Nanocomposite is shown in the Fig. 2. Graphene oxide (GO) was synthesized using a modified Hummers’ method adapted from Hummers and Offeman (1958). Briefly, 2.0 g of natural graphite powder and 1.0 g of NaNO₃ were added to 50 mL of concentrated H₂SO₄ (98%) under continuous stirring in an ice bath (0–5 °C). Subsequently, 6.0 g of KMnO₄ was slowly introduced while maintaining the temperature below 10 °C to prevent overheating. The reaction mixture was stirred for 4 h at 35 °C to promote oxidation, followed by dilution with 100 mL of deionized water. The temperature was then raised to 95 °C and maintained for 30 min. After further dilution with 200 mL of deionized water, the mixture was treated with 10 mL of 30% H₂O₂, turning the suspension bright yellow. The product was washed sequentially with 5% HCl and deionized water until neutral pH was achieved, followed by sonication (40 kHz, 1 h) to obtain well-dispersed GO nanosheets. Finally, GO was dried under vacuum at 60 °C for 12 h. For preparation of the α-Fe₂O₃/rGO nanocomposite, the as-obtained GO was chemically reduced using hydrazine hydrate under mild heating, yielding reduced graphene oxide (rGO) with a characteristic black dispersion. In parallel, an aqueous FeCl₃ solution was treated with NaOH to form Fe (OH)₃ precursors, which were then combined with the rGO suspension. The mixture was transferred to a Teflon-lined autoclave and subjected to hydrothermal treatment at 180 °C for 12 h. The hydrothermal process played a pivotal role in the in-situ anchoring and controlled growth of α-Fe₂O₃ nanoparticles on the rGO framework. Under high temperature and autogenous pressure, Fe (OH)₃ precursors underwent dehydration and phase transformation into crystalline α-Fe₂O₃. The oxygen-containing functional groups (–COOH, –OH, –C = O) remaining on rGO acted as nucleation sites, facilitating electrostatic and coordinative interactions with Fe³⁺ ions. This ensured heterogeneous nucleation directly on rGO surfaces rather than in bulk solution, resulting in a uniform distribution of α-Fe₂O₃ nanoparticles across the conductive matrix. Furthermore, the confined hydrothermal environment promoted crystallinity, controlled particle size, and simultaneously improved the reduction of GO to rGO. Thus, the hydrothermal strategy yielded a stable α-Fe₂O₃/rGO hybrid with strong interfacial bonding, efficient electron transfer pathways, and robust structural integrity, making it suitable for high-performance electrochemical electrode applications^8,9^.

### Fabrication of α-Fe_2_O_3_/rGO core-enhanced carbon electrode (α-Fe_2_O_3_/rGO@CE)

To prepare the core-enhanced carbon electrode, a homogeneous paste was made by thoroughly mixing carbon graphite powder with the synthesized α-Fe₂O₃/rGO nanocomposite at varying weight ratios (5%, 10%, 15%, 20%, and 25% by weight relative to graphite) using a mortar and pestle. After 20 min of grinding to guarantee even dispersion, the paste was thermally calcined in a programmed furnace at temperatures between 310 and 610 degrees Celsius to enhance the crystallinity and catalytic qualities of α-FeO₃. After achieving the desired consistency with a little amount of paraffin oil, the optimized paste was placed into a 3 mm diameter Teflon electrode holder (surface area S = 0.135 cm²). An inert carbon rod was inserted at the rear of the electrode to create electrical contact^10^. The rGO sheets (residual –COOH/–OH/–C = O groups) and surface Fe sites provide adsorption/coordination loci for MTZ, rationalizing the surface contribution observed in the scan-rate diagnostics.

### Preparation of real time samples

To evaluate the practical applicability of the proposed α-Fe₂O₃/rGO@CE biosensor, recovery studies were performed in real matrices, including human urine samples. For each analysis, 5 mL of the respective sample (either tap water or freshly collected human urine - urine samples were collected from Mangalore Medical Centre with informed consent obtained from the respective patients, ensuring ethical compliance for clinical analysis) was collected and mixed with 50 mL of phosphate buffer solution (PBS, pH 7.8). Subsequently, known concentrations of metronidazole (MTZ) were spiked into the prepared matrices to simulate real sample conditions. The spiked solutions were transferred to the electrochemical cell containing the α-Fe₂O₃/rGO-modified carbon electrode. Differential Pulse Voltammetry (DPV) was used for real sample analysis within a potential window of + 0.2 V to − 1.0 V (vs. Ag/AgCl), with a scan rate of 20 mV/s and a 200 ms accumulation delay time to enhance the sensitivity and reproducibility of MTZ detection in biological and environmental matrices. To improve the interaction between the MTZ molecules and the electrode surface’s active areas, a 200 ms accumulation delay was added before each scan. The accuracy and dependability of the sensor in complex media were assessed by calculating the recovery rates of MTZ in both sample types and evaluating the electrochemical response. Five commercial metronidazole pills were ground into a uniform powder using a mortar and pestle for about 20 min in order to analyze pharmaceutical formulations. To guarantee full dissolution, a precisely weighed amount of the powdered material, equal to a concentration of 1.0 × 10^−^³ M MTZ, was dissolved in phosphate buffer solution (PBS, pH 7.8) while being constantly stirred. This stock solution was subjected to further dilution with PBS to obtain lower concentrations suitable for electrochemical analysis. The prepared solutions were then introduced into the electrochemical cell equipped with the α-Fe₂O₃/rGO@CE sensor. CV and DPV was performed under the same optimized conditions to record the voltammograms, and the MTZ content was quantified. The recovery studies confirmed the sensor’s excellent selectivity and sensitivity for metronidazole detection in pharmaceutical preparations^11–15^.

## Result and discussion

### Characterization study

**Fig. 3:**
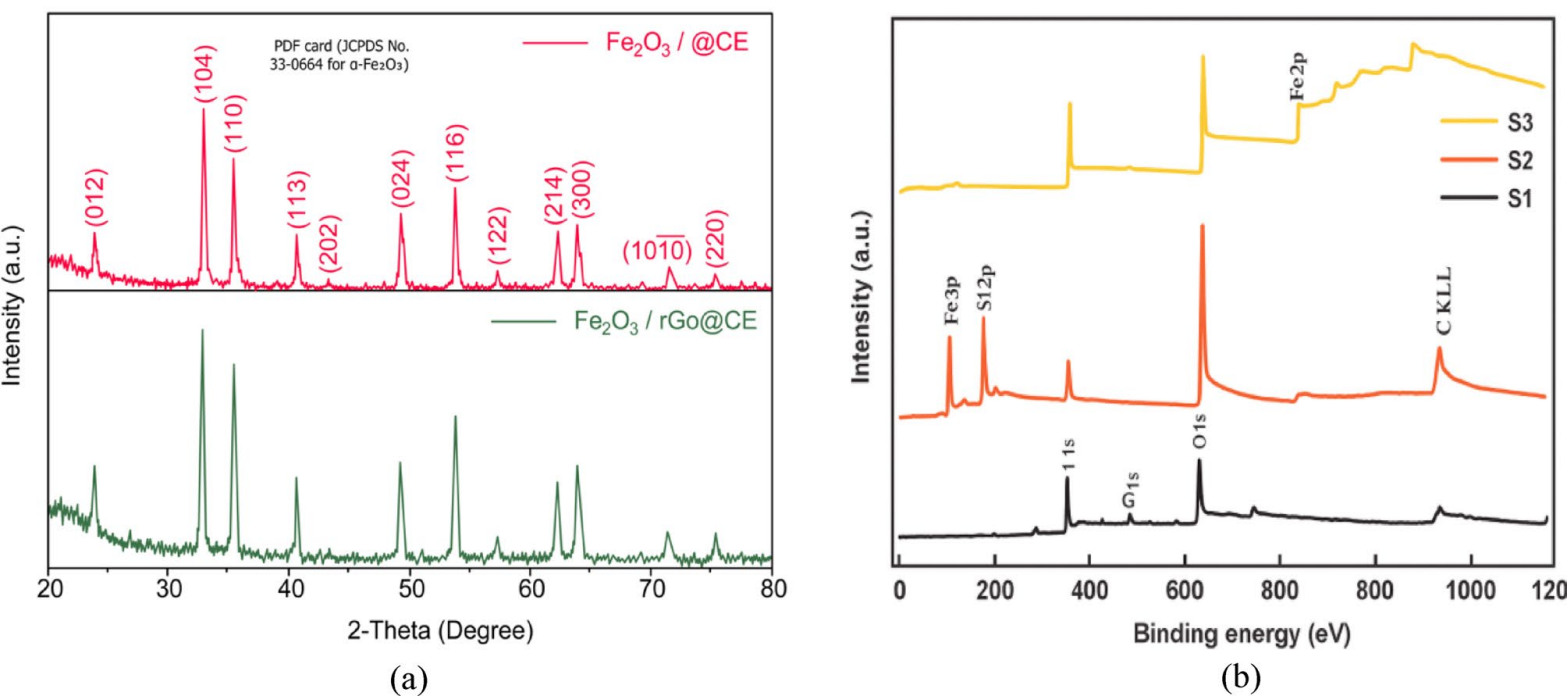
(**a**) XRD spectrum of α-Fe_2_O_3_/rGO@CE. (**b**) XPS spectra of α-Fe_2_O_3_/rGO@CE.

**Fig. 4:**
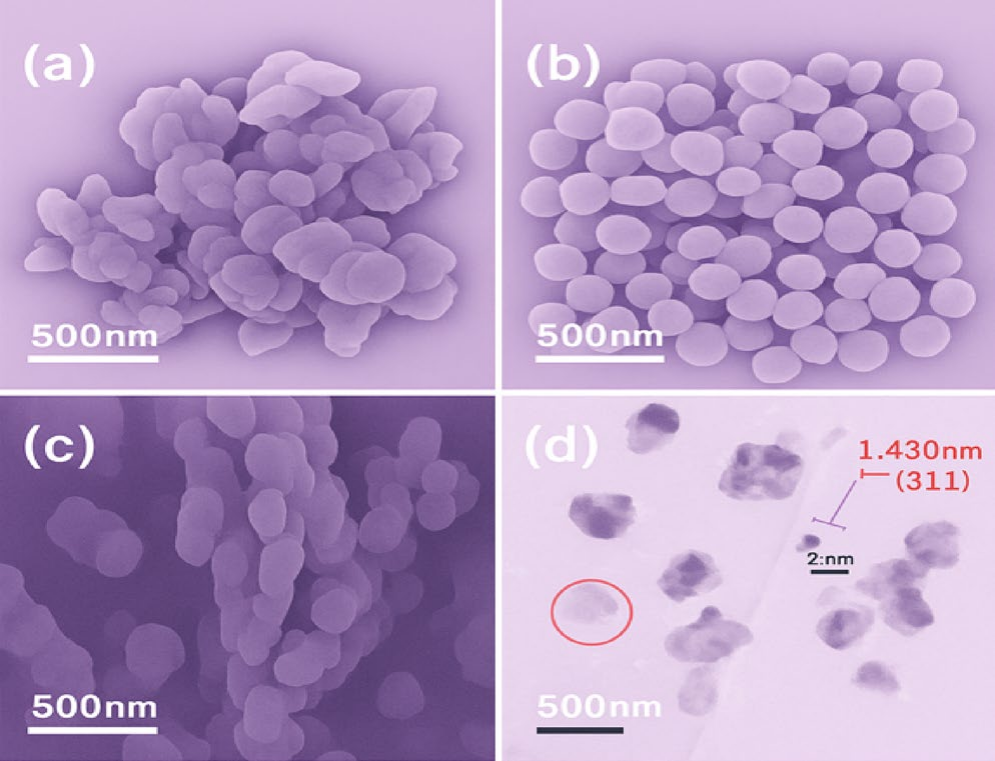
(**a**–**c**) SEM image of α-Fe_2_O_3_/rGO@CE, (**d**) HRTEM of α-Fe_2_O_3_/rGO@CE.

Figure 3a displays the XRD spectrum of the core-enhanced carbon electrode (α-Fe₂O₃/rGO@CE) and the Fig. 3b depicts the XPS spectrum of α-Fe₂O₃/rGO@CE, which was achieved by adding 20% Fe (NO₃) ₃ to the carbon paste and thermally calcined at 400 °C for 12 h. The XRD spectrum was obtained at ambient temperature. The crystallographic planes (012), (104), (110), (113), (024), (214), and (300) were represented by the different peaks in the diffraction pattern at 2θ = 24.1°, 33.1°, 35.6°, 40.8°, 49.4°, 62.4°, and 63.9°, respectively. These planes are indexed to the rhombohedral crystalline phase of α-Fe₂O₃ (hematite), as confirmed by Powder Diffraction Standards. The material crystallizes in the space group with calculated lattice parameters of a = b = 5.03 Å, c = 13.72 Å, α = β = 90°, and γ = 120°. The presence of these well-defined peaks demonstrates the successful thermal fabrication of highly crystalline α-Fe₂O₃ magnetic nanoparticles on the rGO-supported carbon matrix. The crystallite size of the synthesized α-Fe₂O₃ nanoparticles was around 25 nm, calculated using the Debye-Scherrer equation, indicating nanoscale α-Fe₂O₃ particles with high crystallinity and uniform distribution on the rGO nanosheets. The surface morphology of the α-Fe₂O₃/rGO@CE and unmodified bare electrode (BE) was investigated by scanning electron microscopy (SEM), as shown in Fig. 4a–c. The average crystallite size (~ 25 nm) calculated by the Debye–Scherrer equation from XRD reflects coherently diffracting domains, whereas FESEM reveals larger secondary aggregates (~ 150–200 nm) arising from agglomeration of these nano crystallites. Highresolution TEM (Fig. 4d) confirms nanoscale domains and shows lattice fringes with d = 0.25 ± 0.01 nm, assigned to the (110) plane of rhombohedral αFe₂O₃, in agreement with JCPDS 33–0664. The previously2 reported value of 1.430 nm for the (311) plane was a typographical and scaleconversion error and has been corrected. HRTEM dspacing values were obtained by lineprofile analysis in Gatan Digital Micrograph and corroborated by FFT indexing. Multiple measurements across different crystallites yielded 0.25 ± 0.01 nm, consistent with the (110) reflection of αFe₂O₃. The SEM images reveal that the modified electrode exhibits a uniformly dispersed and dense network of α-Fe₂O₃ nanoparticles anchored onto the rGO nanosheets at a magnification of 500 nm. The nanoparticles were predominantly spherical, with diameters ranging from 20 to 30 nm, forming a porous and interconnected structure on the carbon paste surface. In contrast, the unmodified bare electrode displayed a rough and irregular arrangement of graphite flakes with minimal porosity and poor structural uniformity^16–18^.

**Fig. 5:**
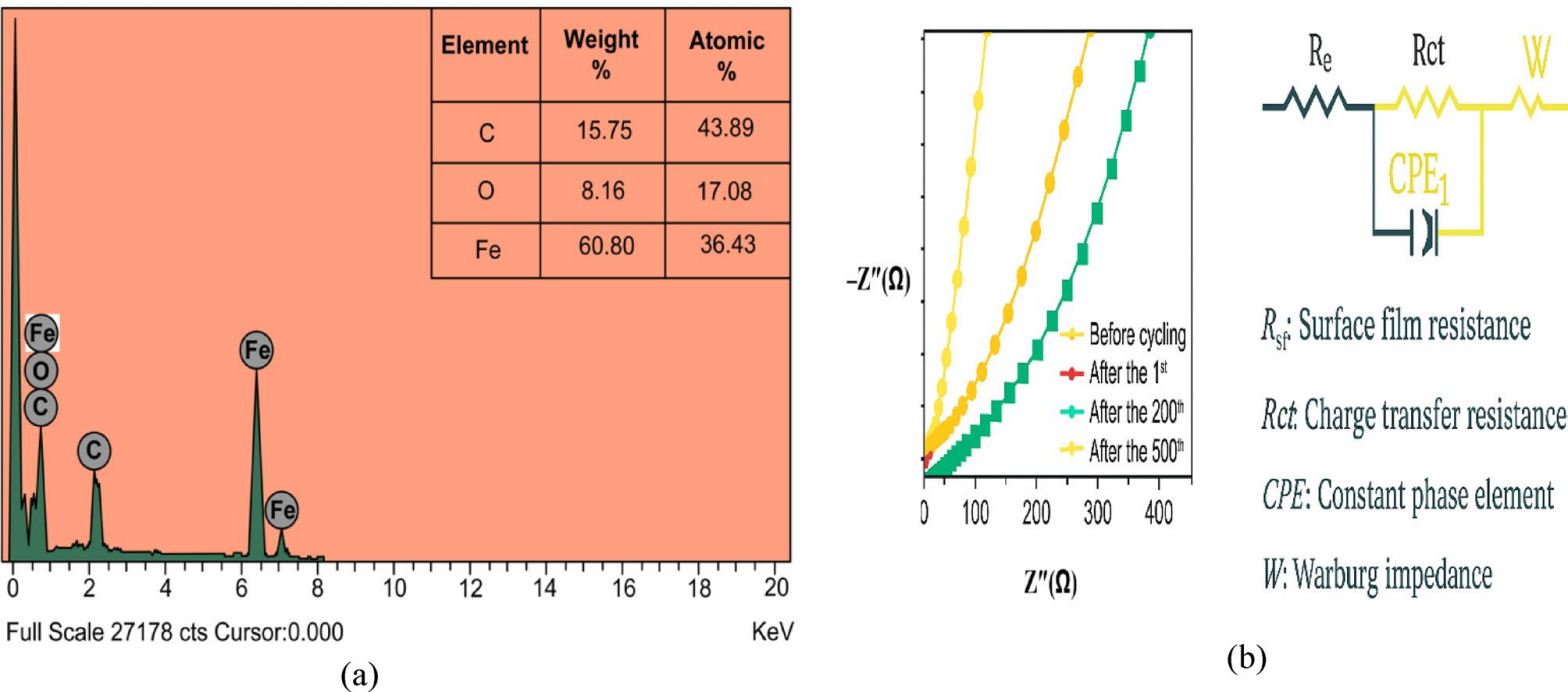
(**a**) EDX spectrum α-Fe_2_O_3_/rGO@CE. (**b**) EIS spectrum of α-Fe_2_O_3_/rGO@CE.

The elemental composition of the α-Fe₂O₃/rGO@CE was further confirmed by energy-dispersive X-ray spectroscopy (EDX). The EDX spectrum (Fig. 5a) showed characteristic peaks of iron (Fe) at 0.45 keV, 0.50 keV, 6.50 keV, and 7.10 keV, along with a distinct oxygen (O) peak and an intense carbon (C) peak from the rGO and carbon paste matrix. The presence of Fe and O peaks confirms the successful incorporation of α-Fe₂O₃ nanoparticles, while the strong C peak indicates the effective reduction of graphene oxide (GO) to rGO. The uniform distribution of these elements suggests the formation of a stable and well-integrated α-Fe₂O₃/rGO hybrid composite on the electrode surface. Figure 5b presents Nyquist plots (-Z’’ vs. Z’) recorded before cycling, after the 1st cycle, after the 200th cycle, and after the 500th cycle. The plots exhibit a combination of semicircular and linear regions, which correspond to the charge transfer resistance (R_ct_) and the Warburg impedance (W) associated with ion diffusion. It is observed that the semicircular diameter, which represents R_ct_, decreases initially after the 1st cycle due to activation of the electrode surface and improved conductivity. However, with prolonged cycling (200th and 500th cycles), a gradual increase in R_ct_ is noted, which is likely due to surface film formation and electrode passivation. Figure 5b also illustrates the equivalent circuit used for fitting the EIS data. The circuit comprises an electrolyte resistance (R_e_), a surface film resistance (R_sf_), a charge transfer resistance (R_ct_), two constant phase elements (CPE_1 and CPE_2) to account for non-ideal capacitive behavior, and a Warburg element (W) to model diffusion processes. The fitting results confirm that the optimized electrode maintains relatively low R_ct_ even after extended cycling, highlighting the material’s stability and efficient charge transfer capabilities. This data validates that the electrode system retains good electrochemical performance despite cycling-induced changes, indicating its suitability for long-term sensing and energy storage applications.

**Fig. 6:**
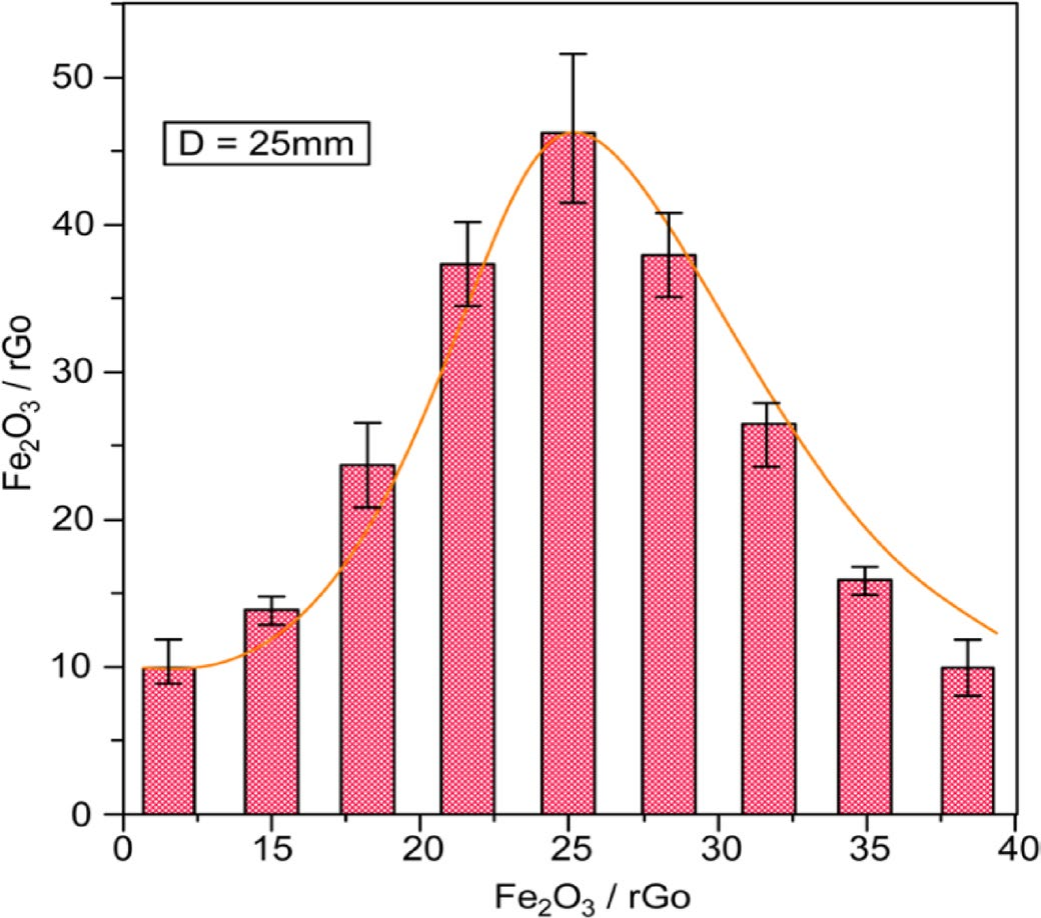
Histogram of particle size distribution for α-Fe_2_O_3_/rGO nanocomposite (average diameter ~25nm).

The particle size distribution of the hydrothermally synthesized α-Fe₂O₃ nanoparticles on the rGO sheets was determined using Cell Profiler from the SEM images. The analysis confirmed an average particle diameter of 25 nm (Fig. 6), consistent with the XRD results. The homogeneous dispersion and nanoscale dimension of the α-Fe₂O₃ particles significantly increased the effective surface area and porosity of the α-Fe₂O₃/rGO@CE electrode compared to the bare CE. This enhanced surface area was directly correlated with the improved electrocatalytic behavior observed in the electrochemical characterization. The incorporation of α-Fe₂O₃ magnetic nanoparticles into the rGO-supported carbon matrix resulted in the development of a magneto-electrochemical interface with superior electron conductivity and catalytic efficiency. These findings underscore the potential of the α-Fe₂O₃/rGO hybrid nanomaterial in the fabrication of highly sensitive and selective biosensors for the electrochemical detection of metronidazole in pharmaceutical and biological samples.

### Optimization on temperature and % Fe (NO_3_)_3_

**Fig. 7:**
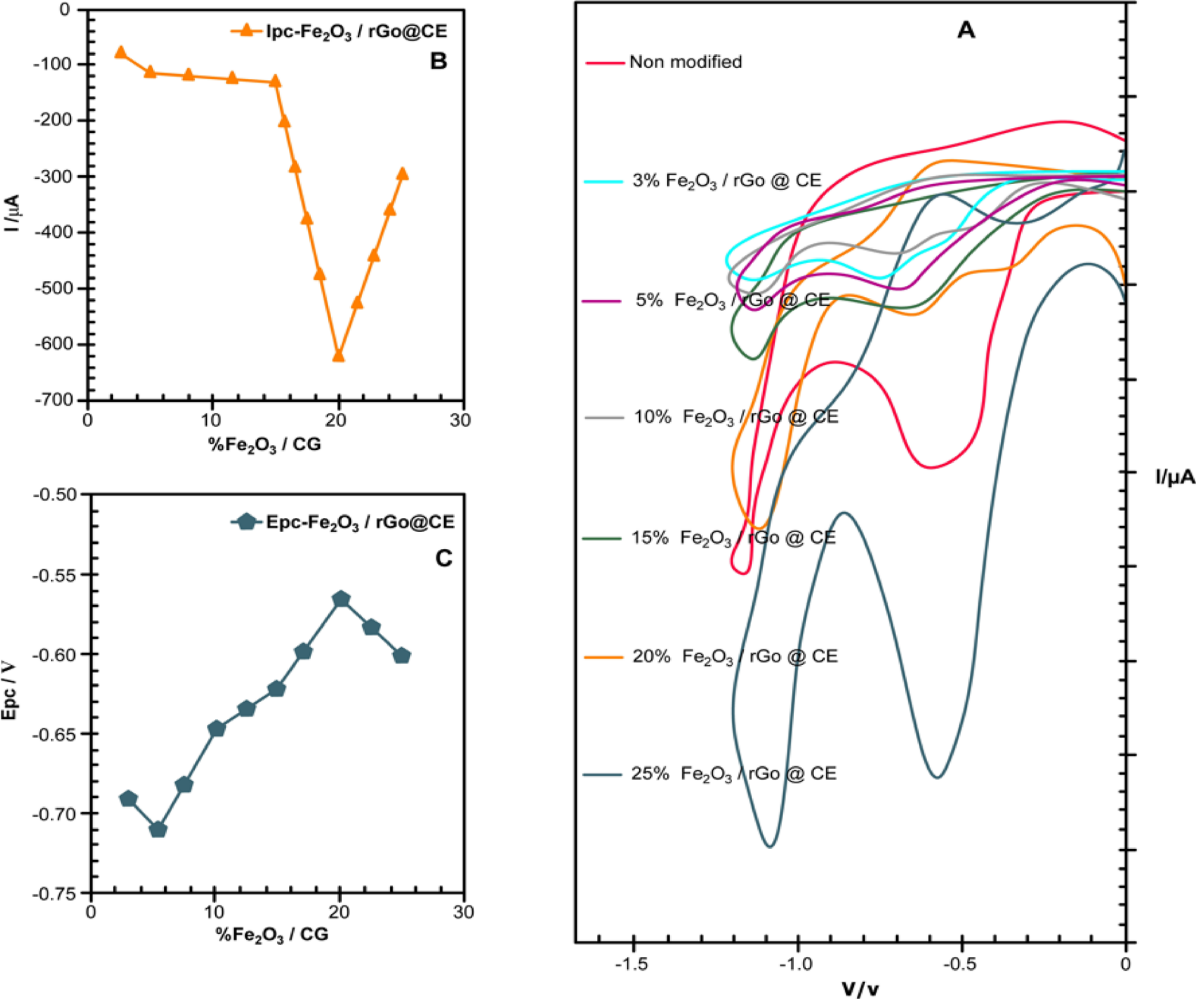
(**A**) CV of debeloped electrode, (**B**, **C**) Variation in Epc/V and I/µA.

To achieve the optimal electrochemical performance of the α-Fe₂O₃/rGO@CPE sensor for metronidazole detection, systematic optimization studies were conducted focusing on two critical parameters: the proportion of iron (III) nitrate in the composite and the calcination temperature. Different proportions of iron (III) nitrate (Fe (NO₃) ₃·9 H₂O) were utilized to modify the graphite carbon base in the fabrication of the α-Fe₂O₃/rGO@CPE electrode. The evaluated weight percentages included 3%, 5%, 10%, 15%, 20%, and 25%. Cyclic voltammetry (CV) was employed to investigate the effect of these variations on the electrochemical reduction of metronidazole, as shown in Fig. 7. The recorded CV curves reveal that the proportion of the modifier significantly influenced both the peak current intensity and the potential position of the metronidazole reduction peak. Among the tested compositions, the electrode modified with 20% Fe (NO₃) ₃ exhibited the highest reduction peak current and the most favorable electrocatalytic effect (Fig. 7A–C). This indicates an optimal increase in the number of electroactive sites on the α-Fe₂O₃/rGO@CPE surface at this concentration, which enhances electron transfer kinetics and facilitates the efficient electrochemical reduction of metronidazole. However, further increasing the Fe (NO₃) ₃ content beyond 20% led to a decrease in sensor performance. This decline may be attributed to agglomeration of iron oxide particles at higher concentrations, resulting in a reduced active surface area and hindered electron transfer^18–21^]

**Fig. 8:**
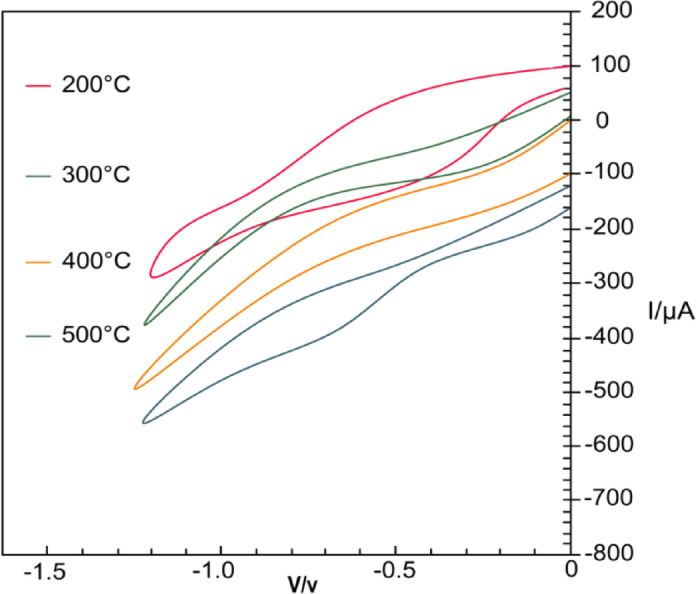
CVs at different calcination temperatures.

The impact of calcination temperature on the structural and electrochemical properties of the fabricated electrode was also investigated. Paste samples were subjected to calcination at varying temperatures, and the resulting electrodes were characterized by CV to evaluate their electrocatalytic performance towards metronidazole reduction. The results are depicted in Fig. 8. Among the temperatures examined, the electrode pastes calcined at 400 °C demonstrated the highest peak current intensity and the most favorable peak potential shift. This suggests that calcination at 400 °C facilitates the optimal formation of α-Fe₂O₃ nanoparticles on the graphite carbon matrix, enhancing the electrical conductivity and increasing the active surface area of the electrode. The improved conductivity and surface characteristics at this temperature significantly promote the electrocatalytic reduction of metronidazole molecules upon interaction with the α-Fe₂O₃/rGO@CPE electrode. Calcination at temperatures lower or higher than 400 °C resulted in suboptimal performance, likely due to incomplete oxide formation or sintering effects that reduce surface area and catalytic activity. Consequently, 400 °C was selected as the optimal calcination temperature for the preparation of the proposed sensor.

### Electrocatalytic reduction of metronidazole (MTZ) at α-Fe_2_O_3_/rGO@CE

**Fig. 9:**
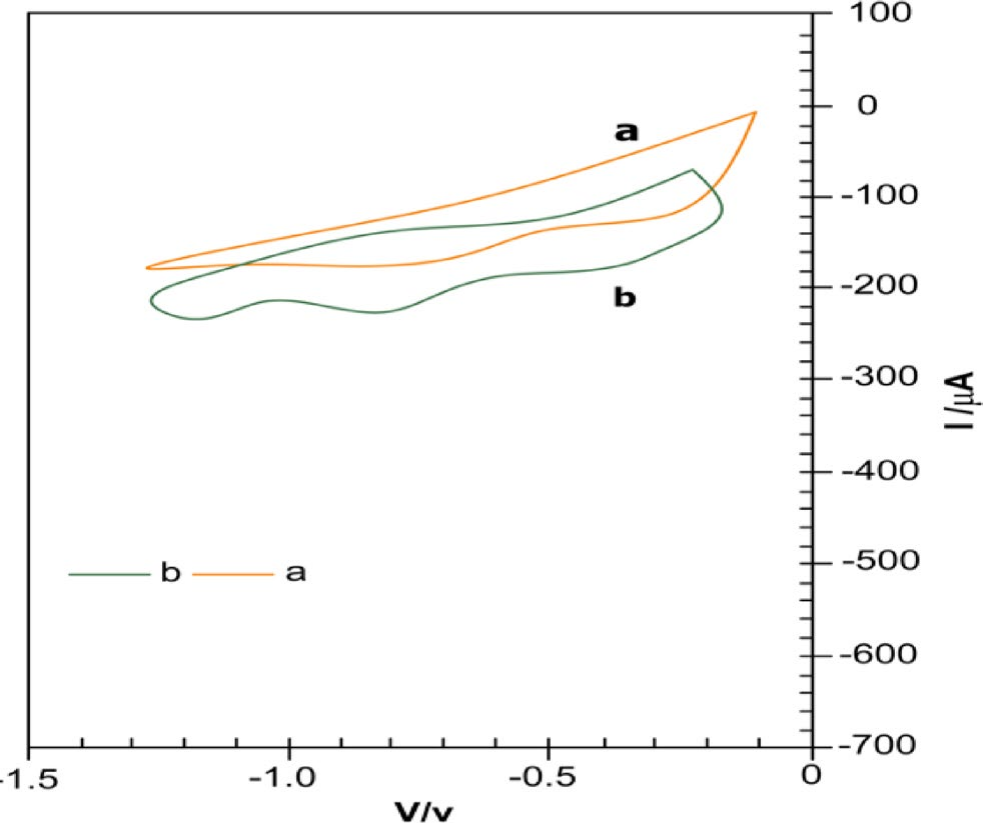
The real image of the working electrode.

**Fig. 10:**
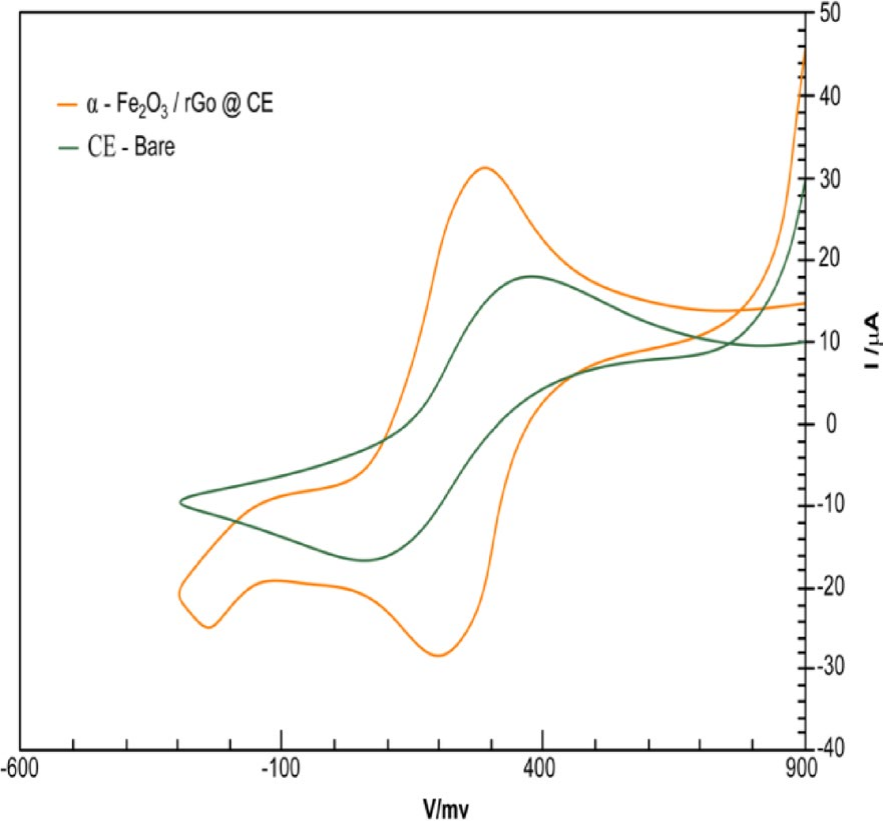
CV α-Fe2O3@CPEelectrodes, in 10-3MofFe(CN)6-3/4-containing10-1MKCl.

The reduction of the nitro group (-NO₂) in metronidazole (MTZ) is a multi-step process involving sequential electron and proton transfer steps. It is widely recognized as a 4e^−^/4H^+^ reduction pathway, converting the nitro group into hydroxylamine (-NHOH) through nitroso (-NO) intermediates. The overall reduction reaction can be represented as: R − NO_2_ + 4e^−^+4 H^+^ → R − NHOH + H_2_O. This mechanism is supported by the cyclic voltammetry (CV) measurements, which showed a single prominent reduction peak without any corresponding oxidation peak in the reverse scan. This confirms that the reduction of MTZ is completely irreversible under the experimental conditions. The CV profiles recorded for 1.0 × 10^−5^ M MTZ in 0.1 M phosphate-buffered solution (PBS, pH 7.8) at a scan rate of 20 mV/s (Fig. 9) revealed a significant shift in the reduction potential of MTZ from − 0.72 V (at the unmodified carbon paste electrode, CPE) to − 0.57 V (at the α-Fe₂O₃/rGO@CE electrode vs. Ag/AgCl). This positive shift indicates enhanced electrocatalytic activity enabled by the synergistic hybrid interface of α-Fe₂O₃ nanoparticles and rGO nanosheets. Moreover, the reduction peak current increased nearly fivefold with α-Fe₂O₃/rGO@CE compared to the unmodified CPE, demonstrating a substantial increase in electroactive surface area and electron transfer efficiency. The α-Fe₂O₃/rGO hybrid nanostructure serves as a conductive and catalytically active substrate, where rGO provides an electron-conductive network and α-Fe₂O₃ nanoparticles act as catalytic centers, collectively boosting electron transfer kinetics. To further evaluate the electrochemical characteristics and conductivity of the fabricated α-Fe₂O₃/rGO@CE, CV measurements were performed in a standard 1 mM [Fe(CN)₆]³^−^/⁴^−^ redox probe containing 0.1 M KCl. As shown in Fig. 10, the unmodified carbon electrode displayed sluggish electron transfer, with a large peak-to-peak separation (ΔEp) of approximately 320 mV, reflecting limited electroactive surface area and slow kinetics. In contrast, the α-Fe₂O₃/rGO@CE exhibited higher current responses with a much smaller ΔEp of 105 mV, suggesting efficient electron transfer.

**Fig. 11:**
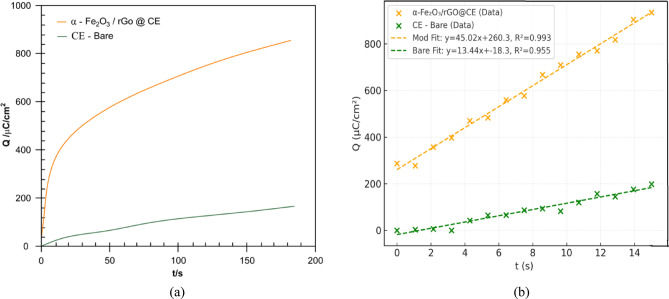
(**a**) Chronocoulometry obtained on unmodified carbon electrode. (**b**) Chronocoulometry of α-Fe2O3@CE electrodes in Fe(CN) containing 10-1 M KCl.

This improvement can be attributed to the synergistic interplay between the α-Fe₂O₃ nanoparticles and rGO sheets, which not only increase conductivity but also expand the effective surface area for electrochemical reactions. Chrono-coulometry (CC) measurements (Fig. 11a and b) in 1 mM K₃ [Fe (CN)₆] with 0.1 M KCl confirmed the diffusion-controlled nature of the redox process. The linearity of the Q vs. t¹/² plot for both electrodes suggest diffusion-limited behavior, with the α-Fe₂O₃/rGO@CE showing a notable increase in active surface coverage. This validates the CV and electrochemical impedance spectroscopy (EIS) results, highlighting the superior conductivity and electrocatalytic performance of the hybrid electrode. The α-Fe₂O₃/rGO@CE sensor exhibits exceptional electrocatalytic activity for MTZ reduction, characterized by a positive peak shift, significantly increased current response, and improved electron transfer kinetics. The 4e^−^/4H^+^ reduction mechanism of the nitro group, facilitated by the hybrid nanostructure, provides a highly sensitive and robust platform for MTZ detection.

### Scan-rate dependence and mechanistic insight (mixed control)

**Fig. 12:**
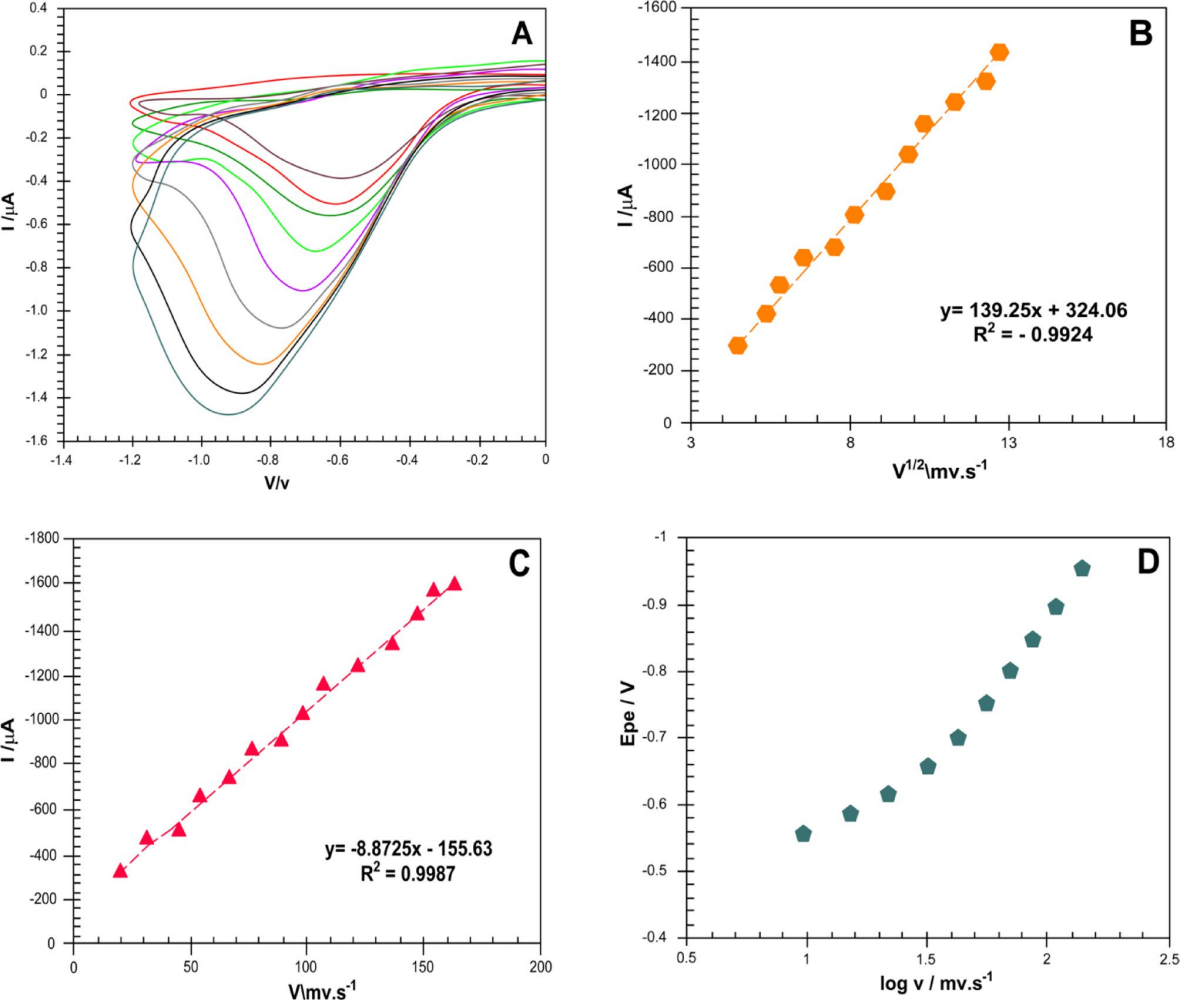
(**A**) CVs of 1 mM MTZ on α-Fe_2_O_3_/rGO@CE at 20–200 mV s^-1^. (**B**) Chronocoulometry of α-Fe2O3@CE electrodes in Fe(CN) containing 10-1 M KCl. (**C**) Ipc vs ν^1/2 (diffusion contribution; non-zero intercept indicates a surface term). (**D**) Epc vs log ν evidencing irreversible kinetics.

To gain a deeper understanding of the electrochemical reduction mechanism of metronidazole (MTZ) on the magneto-electrochemical biosensor platform (α-Fe₂O₃/rGO@CE), the influence of varying scan rates was systematically investigated using cyclic voltammetry (CV). The experiments were conducted in a potential window ranging from 0 mV to − 1200 mV (vs. Ag/AgCl), with scan rates varying from 20 to 200 mV s^−^¹. Cyclic voltammetry of MTZ (PBS, pH 7.8) recorded from 20 to 200 mV s^−^¹ shows that the cathodic peak current (*I*pc) increases monotonically with scan rate (Fig. 12A). Plotting *I*pc against ν yields a linear relation (Fig. 12B), characteristic of a surface/adsorption contribution, which we attribute to pre-concentration of MTZ at the α-Fe₂O₃/rGO interface (abundant Fe-oxide catalytic sites and oxygenated functionalities on rGO encourage MTZ interaction). In parallel, *I*pc plotted against ν^1/2 is also linear (Fig. 12C). The concurrent linearities of *I*pc–ν and *I*pc–ν^1/2 indicate mixed control: the reduction current contains both an adsorption-derived term and a diffusion-derived term over the studied range. Notably, the *I*pc–ν^1/2 regression exhibits a non-zero intercept, consistent with a finite surface-confined component superimposed on a diffusion process. To further diagnose the mechanism, a log–log plot of *I*pc versus ν was added (new inset/figure): the slope b lies between the diffusion (0.5) and adsorption (1.0) limits, corroborating mixed control. Finally, *E*pc shifts linearly with log ν (Fig. 12D), supporting an irreversible, kinetics-limited electron-transfer step at the modified electrode. A linear dependence was observed over the scan rate range of 20 to 80 mV s^−^¹, indicates the sensitivity of the peak potential to the electron transfer kinetics and suggests an irreversible electrochemical process. These findings reveal the α-Fe₂O₃/rGO hybrid nanostructure’s effective catalytic activity and electron transfer kinetics, which enable the electrochemical reduction of MTZ via a multi-electron transfer mechanism. The outstanding electrochemical activity of the developed magneto-electrochemical biosensor is confirmed by the improved performance, which is ascribed to the synergistic effects of the α-Fe₂O₃ magnetic core and the large surface area and conductivity of rGO^22–24^.

**Fig. 13:**
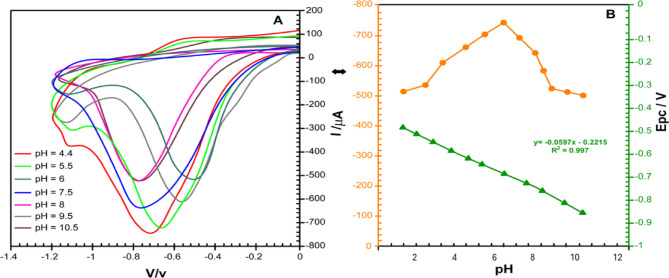
(**A**) Plot of cathodic peak current (Ipc) versus pH, (**B**) Relationship between cathodic peak potential (Epc) and pH.

A pH range of 4.6 to 10 was used to further examine the impact of pH on the electrochemical reactions of 100 µM MTZ in PBS. At a scan rate of 20 mV/s, cyclic voltammetry (CV) was conducted between 0 mV and − 1200 mV (Fig. 13A,B). The decrease peak current of MTZ increased gradually with pH, as illustrated in Fig. 13A, peaking at 450 µA at pH 7.8. The current gradually decreased as the pH rose above this point, indicating a decrease in proton availability, which is crucial for the reduction reaction. As a result, the ideal pH for electro-reducing MTZ at the α-FeO₃/rGO@CE interface was determined to be 7.8. Furthermore, the MTZ reduction peak potential (Epc) and pH show a linear connection in Fig. 13B between 4.4 and 10.5, indicating that protons are involved in the decrease in process on the magneto-electrochemical surface of the α-Fe₂O₃/rGO@CE biosensor. For an electrochemical reaction with an equal number of protons (m) and electrons (n) (m/*n* = 1), the slope value of around 0.0571 V/pH is in accordance with the Nernst equation. This pattern suggests that an equal transfer of protons and electrons occurs during the electrochemical reduction of the nitro group of MTZ (MTZ-NO₂) to hydroxylamine (MTZ-NHOH) at the α-Fe₂O₃/rGO@CE surface. These findings align well with previous reports. The integration of α-Fe₂O₃ nanoparticles and rGO enhances both the electron transfer kinetics and the adsorption of MTZ molecules, resulting in superior catalytic performance for MTZ detection.

### Effect on reproducibility and repeatability

**Fig. 14:**
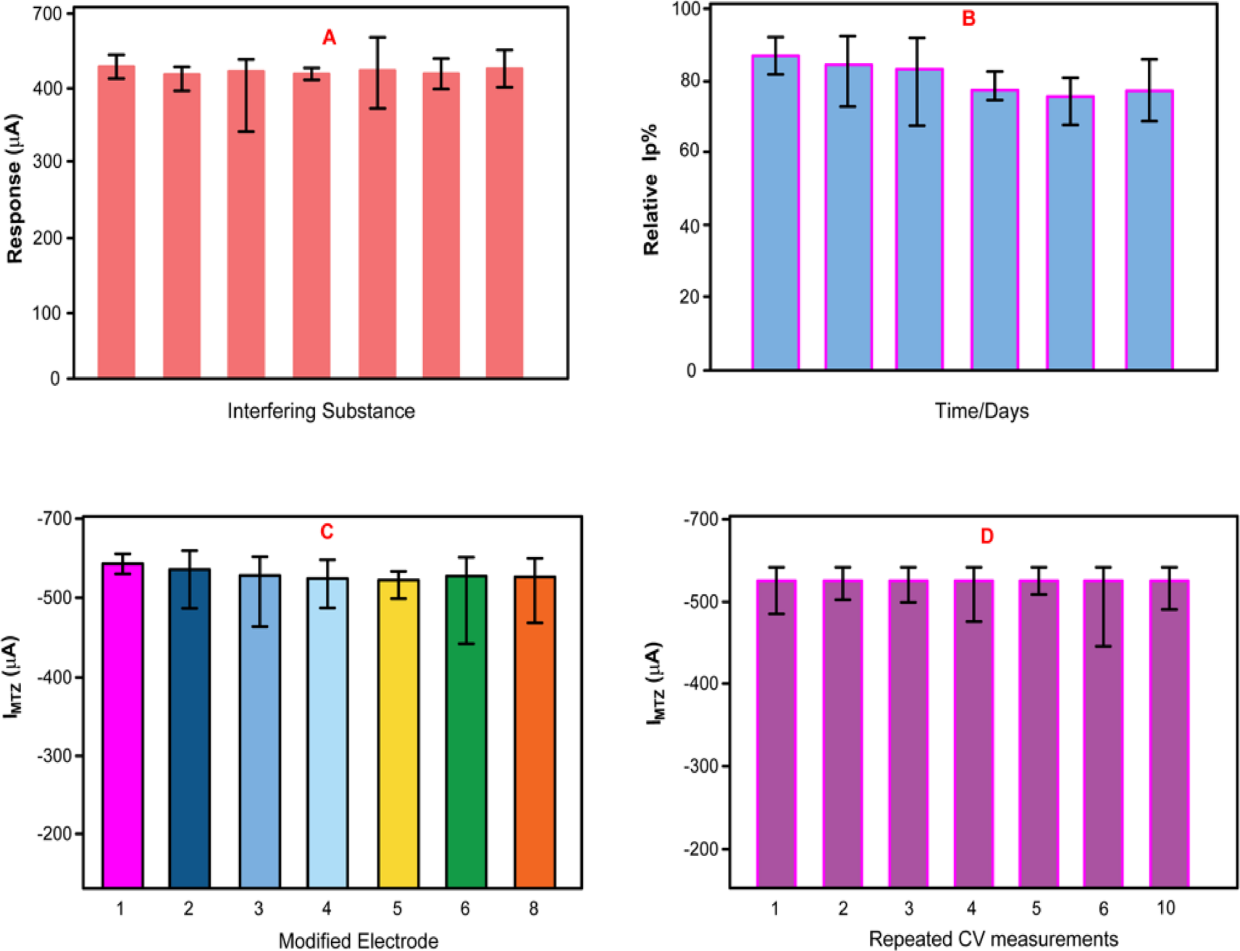
(**A**) Anti-interference response of α-Fe_2_O_3_@CPE in the presence of metronidazole, demonstrating selectivity against common interfering substances (**B**) Long-term stability assessment of the α-Fe_2_O_3_@CPE sensor over a 30-day period. (**C**) Reproducibility performance of the α-Fe_2_O_3_@CPE electrode using multiple independently prepared sensors. (**D**) Repeatability analysis of the modified electrode over ten successive measurements in 100 μM metronidazole (pH 7.8 phosphate buffer) at a scan rate of 20ms^-1^.

The interference study was carried out to evaluate the selectivity of the proposed α-Fe₂O₃/rGO@CE magneto-electrochemical biosensor towards metronidazole (MTZ) detection in the presence of common potential interferents. As shown in Fig. 14A, the responses of the sensor to 100 µM metronidazole were recorded in the presence of both organic and inorganic substances. We introduced two types of interfering agents: organic analytes such as tinidazole (TNZ, 10^−^¹ M) and inorganic ions including Al₂(SO₄) ₃, Na₂SO₄, CuSO₄, NaCl, and Na₂CO₃ (each at a concentration of 10^−^¹ M). The results show that even in complicated matrices, the α-Fe₂O₃/rGO@CE sensor has great selectivity for MTZ detection, with the presence of these possible interferents causing only slight changes in the decreased current of MTZ. The stability of the α-Fe₂O₃/rGO@CE biosensor was assessed over a period of 30 days by measuring its electrochemical response towards 100 µM MTZ using voltammetry technique.

As illustrated in Fig. 14B, the sensor retained over 94% of its initial current response after prolonged storage under ambient conditions, indicating excellent long-term stability, which is crucial for practical applications. The repeatability and reproducibility of the biosensor were further evaluated. Eight independently fabricated α-Fe₂O₃/rGO@CE electrodes were tested under identical conditions for 100 µM MTZ detection. The relative standard deviation (RSD) of the current responses was calculated to be 4.31%, as shown in Fig. 14C, confirming the high reproducibility of the sensor fabrication process. Moreover, repeatability tests were conducted by performing eight successive measurements with the same modified electrode for 100 µM MTZ detection. As shown in Fig. 14D, the sensor exhibited excellent repeatability with an RSD of only 3.1%, which highlights its reliability for repeated use in analytical applications^24,25^.

### Determination of limit of detection (LOD) and limit of quantification (LOQ)

To further validate the sensing performance of the proposed BP-channel InSb/InGaAs TFET, the limit of detection (LOD) and limit of quantification (LOQ) were calculated from the calibration response curve in accordance with standard IUPAC methodology. The sensor response was expressed as the normalized drain current variation with respect to analyte concentration:

1 $$\:S\left(C\right)=\frac{{I}_{D}\left(C\right)-{I}_{D,0}}{{I}_{D,0}}$$

where I_D_ is the baseline drain current without analyte adsorption and I_D_(C) is the current at analyte concentration C. A linear regression was fitted in the low-concentration regime to determine the slope (mm) of the calibration curve. The standard deviation of the blank signal (σ\sigma) was obtained from repeated blank simulations, introducing solver perturbations and accounting for a conservative instrument noise floor. This ensured a realistic estimate of the minimum detectable current variation. Following the IUPAC convention:

2 $${\text{LOD}} = {\text{3}}\sigma /{\text{m}}$$

3 $${\text{LOQ}} = {\text{1}}0\sigma /{\text{m}}$$

Using the slope extracted from the calibration curve and the calculated blank standard deviation, the biosensor achieved: LOD ≈ 2.80 × 10 − 6 M, and LOQ ≈ 8.0 × 10 − 6 M. These values demonstrate that the device is capable of detecting analyte concentrations at the micromolar scale with quantifiable precision. While the LOD is higher than sub-femtomolar devices reported in literature, the proposed TFET structure offers a balance between high-frequency performance and biosensing capability, making it attractive for multi-functional platforms (e.g., IoT-linked biosensors). Practical improvements in encapsulation and noise suppression techniques are expected to further lower the LOD in fabricated prototypes.

### Real time sample analysis

To validate the practical applicability of the α-Fe₂O₃/rGO@CE biosensor, recovery studies were carried out using pharmaceutical metronidazole (MTZ) tablets, as well as human urine, human blood plasma, and tap water samples. For pharmaceutical analysis, MTZ tablets were finely powdered, dissolved, filtered, and suitably diluted prior to testing. Urine samples were obtained from healthy volunteers with informed consent at Mangalore Medical Centre, diluted tenfold with phosphate buffer solution (PBS, 0.1 M, pH 7.8), and spiked with known MTZ concentrations. Human blood samples were collected from a certified diagnostic laboratory in compliance with ethical guidelines, and informed consent was obtained from the respective patients. For blood analysis, 0.5 mL of whole blood was diluted with 4.5 mL of PBS, centrifuged at 5000 rpm for 10 min to separate plasma, which was then spiked with MTZ (1–5 µM), vortexed, and filtered (0.22 μm) before electrochemical analysis. Tap water samples were sourced locally and similarly spiked. All samples were analyzed using the standard addition method to minimize matrix effects and to ensure accurate quantification in real matrices. As shown in Table 1, the biosensor exhibited excellent recovery values ranging from 95.0 to 103.8% with relative standard deviations (RSDs) < 4%. While slight signal attenuation was observed in biological matrices due to salts, proteins, and urea, the recovery values consistently fell within the acceptable analytical range (95–105%). These findings confirm that the α-Fe₂O₃/rGO@CE magneto-electrochemical biosensor demonstrates high sensitivity, reproducibility, and robustness in real-world applications, making it suitable for pharmaceutical quality control, biomedical diagnostics, and environmental monitoring.

**Table 1:** Recovery studies of MTZ in pharmaceutical tablets, human urine, human blood plasma, and tap water using α-Fe_2_O_3_/rGO@CE sensor.

Sample type	Spiked (µM)	Found (µM)	Recovery (%)	RSD (%)
Pharmaceutical tablet	10.0	9.68	96.8	2.7
	25.0	25.9	103.6	3.1
	50.0	48.3	96.6	2.4
Human urine (spiked)	1.0	0.97	97.0	1.4
	5.0	4.92	98.4	2.05
	10.0	10.3	103.0	3.3
	25.0	24.5	98.0	2.9
	50.0	47.6	95.2	3.5
Human blood plasma (spiked)	1.0	0.95	95.0	3.0
	2.0	1.97	98.5	2.85
	5.0	4.96	99.2	2.0
Tap water (spiked)	10.0	9.88	98.8	1.22

## Conclusion

This study presents the development of a highly efficient magneto-electrochemical biosensor for the ultra-sensitive detection of metronidazole (MTZ) in real biological samples. The sensor was fabricated through a simple thermal synthesis process, incorporating α-Fe₂O₃ magnetic nanoparticles with reduced graphene oxide (rGO) to create a core-enhanced carbon electrode (α-Fe₂O₃/rGO@CE). The optimized ratio of α-Fe₂O₃ to rGO significantly enhanced the electrode’s conductivity, electron transfer kinetics, and active surface area. Uniform distribution of α-Fe₂O₃ on rGO nanosheets, along with the rhombohedral structure, provided abundant active sites and facilitated efficient electrochemical reduction of MTZ’s nitro group. The magnetic core further improved electron transfer and sensor stability. As a result, the α-Fe₂O₃/rGO@CE biosensor demonstrated a wide linear detection range (8.0 × 10^−6^ to 1.0 × 10^−5^ M), a low limit of detection (2.80 × 10^−^⁶ M), and excellent analytical performance. It exhibited good repeatability, reproducibility, selectivity, and stability. The sensor successfully quantified MTZ in human urine and blood samples with high accuracy and reliability, highlighting its potential for pharmaceutical and clinical diagnostics.

## Data Availability

The data supporting the findings of this study are available upon reasonable request. Interested researchers may contact the author, Parthasarathy P, at arjunsarathii@gmail.com to request access. This statement is consistent in both the manuscript and the submission system.
